# The mpox epidemic is not over: Reducing disproportionate burden in Africa and persistent global risk require a sustained response

**DOI:** 10.1371/journal.pmed.1004893

**Published:** 2026-02-03

**Authors:** Dieudonné Mwamba Kazadi, Rosamund F. Lewis, Pierre Akilimali, Danny Kalala, Maria Van Kerkhove, Chikwe Ihekweazu

**Affiliations:** 1 Institut National de Santé Publique, Ministère de la Santé publique, de l’Hygiène et de la Prévoyance sociale, Kinshasa, République Démocratique du Congo; 2 École de Santé Publique de Kinshasa, Faculté de Médecine, Université de Kinshasa, Kinshasa, République Démocratique du Congo; 3 Health Emergencies Preparedness and Response Programme, World Health Organization, Geneva, Switzerland

## Abstract

The 2022 mpox outbreak quickly made headlines and galvanized global attention; but the epidemic is not over. In this Perspective, Rosamund Lewis and colleagues highlight ongoing mpox outbreaks across Africa and the world, and advocate for a sustained global response that moves beyond reactive measures.

## Teaser

While global interest in mpox may be waning, outbreaks, illness, and death continue across Africa and the world. Ending transmission requires a sustained global response that moves beyond reactive measures.

Mpox has been recognized in Africa since 1970, with ever-rising numbers of reported cases [1]; yet global attention was only galvanized in 2022, when an mpox epidemic suddenly emerged and quickly spread around the world [2]. Mpox is a viral infection which causes painful skin and mucosal lesions, fever, headache, back pain, and often (first noted since 2017) serious genital injury [2]. While most people fully recover, mpox can lead to secondary infections, other complications, and death. People living with HIV are at higher risk of acquiring mpox and, if immune compromised, of experiencing severe disease [1,2]. A coordinated global response, led by the World Health Organization (WHO), national authorities, and affected communities, controlled the mpox epidemic due to clade IIb monkeypox virus (MPXV), with 94,576 confirmed cases and 183 deaths reported in 120 countries by the end of 2023. At the same time, mpox epidemics linked to clade I MPXV in the Democratic Republic of the Congo (DRC) were gathering steam and moving beyond historically affected areas [3]. Initially, laboratory confirmation in the country was limited to fewer than 9% of suspected cases, and mpox vaccines—licensed and used in high-income countries since 2022—were unavailable in Africa [3].

While clade IIb MPXV continued to circulate globally, clade I of the virus—long considered to be more virulent—was gaining ground, with a new strain (clade Ib) spreading efficiently through heterosexual networks in Africa [4]. The synchronous reporting of clade Ib in four East African countries in July 2024 finally injected a new sense of urgency into the mpox response in Africa [5]. In 2025 alone, with outbreaks expanding and laboratory capacity improving, 28 countries in Africa reported 90% (42,942 of 47,980) of mpox cases confirmed globally (all clades) and 96% of deaths (192 of 201) (31 October). The DRC reported 22,208 confirmed cases in 2025 to the end of November, with over 1,000 suspected and confirmed cases still reported each week [6].

Rapidly emerging multi-country mpox outbreaks have thus twice been declared a Public Health Emergency of International Concern by the Director-General of WHO under the International Health Regulations (2005) (IHR). In 2024, the Africa Centres for Disease Control and Prevention (Africa CDC) also declared the upsurge of mpox in Africa to be a Public Health Emergency of Continental Security. These declarations served to raise awareness, galvanize public health response, and channel new research towards this under-recognized, under-resourced threat. Globally (and particularly in Africa), calls to action have been issued [7] and response capacity for mpox has been built or strengthened, with lessons learned in high- and low-income settings. Responses at global and national level have highlighted the importance of core public health measures: Boosting surveillance and access to diagnostics, training and capacity-building for all interventions, and ensuring the central role of risk communication and community engagement [8,9]. National regulatory authorities authorized emergency use of mpox vaccines in Nigeria in August 2023 (MVA-BN) and in the DRC in June 2024 (both MVA-BN and LC16 vaccines). The WHO and Africa CDC emergency declarations, however, ultimately catalyzed efforts to facilitate mpox vaccination across Africa. WHO prequalification and emergency use listing for mpox vaccines and diagnostics (beginning 13 September 2024) provided countries assurance of product quality and safety in emergencies. From late September to early December 2024, eight additional African countries had authorized emergency mpox vaccine use and three had begun vaccination, with a further eight authorizations and 11 vaccination starts in 2025. With 5 million mpox vaccine doses delivered to 16 countries in Africa, 2 million people (including 400,000 children) have been vaccinated: 1.2 million with MVA-BN in 14 countries (including the DRC) and 800,000 with LC16 vaccine (DRC only) [6]. New partnerships have deepened necessary collaboration and integration between emergency response and HIV/STI control programs, crucial to reaching people living with HIV [2,8]. Even as WHO and Africa CDC piloted joint incident management for the emergency, close coordination among all partners, including UNICEF, Gavi, and others, has been essential to advancing the mpox response.

Health authorities and communities are thus now well-positioned to respond to mpox outbreaks and these successes carry broader implications for other emerging diseases. Yet, the response is being undermined by funding shortfalls, overstretched health systems, persistent stigma, and competing priorities, threatening capacity to sustain essential interventions—particularly access to vaccines and diagnostics. The public health impact of mpox in endemic areas remains underappreciated, where children are most affected, case fatality is higher, access to quality care is limited, and resources are strained by concurrent outbreaks [1,3]. Viral genome sequencing reveals ongoing zoonotic spillover from a yet unknown animal reservoir [10], and new virus strains will continue to emerge from endemic as well as non-endemic areas, fueling transmission and posing global health security risks.

With secondary attack rates consistently higher among sexual contacts than among family members [11], mpox will continue to find efficient transmission routes. Ongoing spread through interconnected commercial and community sexual networks will sustain the epidemic, reaching people living with HIV whose access to antiretroviral therapeutics is also threatened by cuts to global aid [12]. Mpox will still have major consequences for displaced persons due to community transmission in crowded conditions, for women experiencing adverse pregnancy outcomes, and for children known to be at higher risk of severe disease [1,3]. It remains imperative to engage with communities, improve understanding of risk, and provide access to preventive measures, including vaccines.

Despite extraordinary national efforts and gains achieved, the future trajectory for mpox control relies on sustained political will at all levels and the financial resources to maintain momentum. This cannot once again be Africa’s burden alone [1]. Under the IHR, WHO has extended the Standing Recommendations on mpox for all Member States, bridging to longer-term strategies to prevent and stop mpox transmission [8]. To safeguard health from poxviruses in future, it is necessary to resource a robust long-term plan that ensures sustainable access to countermeasures for Africa. Research and response capacity must still be expanded together with national authorities and local communities.

Although substantial progress has been made, mpox outbreaks remain a serious and urgent global health concern. The resurgence of cases in Africa (particularly in the DRC) [1–6], community transmission of clade Ib MPXV in other regions [6], continued transmission of clade IIb globally [6], and recent emergence of the first documented clade I/clade II recombinant strain of MPXV [13] all underscore the need for sustained, coordinated, and equitable action (see Box 1). These efforts will also strengthen the prevention of other poxvirus-related diseases, such as cowpox in Europe, buffalopox in India, vaccinia in Brazil, and borealpox in the Arctic, any of which could evolve to threaten human health more widely. By acting collectively, we can stay ahead of those risks while advancing mpox prevention and control.

**Box 1. Key priorities for sustained and equitable global and national action on mpox**

Action for sustained and equitable mpox prevention and response must include strategies to:

- Strengthen national public health institutes to drive integrated mpox surveillance, prevention, and response activities, and ensure coordination of multi-partner collaboration through national and sub-national Emergency Operations Centres [8]
- Integrate prevention and response interventions to reduce sexual transmission, support trusted care providers, maintain high-quality clinical care, protect marginalized populations and vulnerable groups, prevent and address stigma, and strengthen engagement with local communities at the heart of the response [8,9]
- Integrate mpox and HIV services (testing, vaccination, and treatment of co-infection) in health facilities and dedicated HIV/STI clinics, especially for key populations [8,12]
- Invest in understanding zoonotic transmission in endemic regions, recognizing that One Health approaches are needed to address all aspects of risk, particularly for children [1,3,8,10]
- Pivot response strategies toward rapid investigation and integrated coordinated action at the first detected case or cluster in an area to prevent outbreak expansion [8,14]
- Strengthen local surveillance and laboratory capacity to detect and monitor outbreaks, including support for affordable and reliable rapid diagnostic tests and transmission studies [1,7,8,14]
- Scale up pre-exposure vaccination to protect people and communities most at risk, in line with recommendations of WHO Strategic Advisory Group of Experts on Immunization (SAGE) [7,8]
- Secure global capacity and predictable financing to meet the need for affordable and accessible mpox vaccines, and to expand production capacity and lower costs, through innovation and technology-sharing [7,8]
- Extend the International Coordinating Group on vaccine provision (ICG) to include mpox to support rapid detection and response with a robust product mix, drive vaccine development, stimulate financial investment, and promote market shaping for additional, lower-cost diagnostics and vaccine options [8]
- Incentivize sustained research for effective and affordable diagnostics, vaccines, optimal clinical care, and development of therapeutics [1,7,8]
- Adapt regulatory procedures for new or improved countermeasures, where feasible and appropriate, to accelerate approvals and sustain emergency use or full market authorization where required for emergency response [7]
- Advance discussion through the World Health Assembly and other fora to secure global commitment to longer-term action.

The sustained transmission of mpox as an unintended consequence of the eradication of smallpox [1] ultimately points the way to the elimination of mpox as a public health problem [8]. We cannot let people suffer from mpox unnecessarily when we can stop it, nor allow the shared legacy of smallpox eradication to fade. All countries should commit to sustained, equitable action, accelerating access to effective and affordable interventions while strengthening health system capacity to integrate mpox diagnostics, vaccines, and rapid response within health services for people at risk. To uphold equity and preparedness, it is time to recognize and sustainably address mpox as a distinct global health priority and renew global commitment to prevention, preparedness and response, including through deliberation at the World Health Assembly.

## Abbreviations

Africa CDC: Africa Centres for Disease Control and Prevention
DRC: Democratic Republic of the Congo
IHR: International Health Regulations (2005)
MPXV: monkeypox virus
WHO: World Health Organization
